# Danshen Attenuates Intervertebral Disc Degeneration via Antioxidation in SD Rats

**DOI:** 10.1155/2020/6660429

**Published:** 2020-12-22

**Authors:** Rongqing Qin, Shouqian Dai, Xing Zhang, Hongpeng Liu, Bing Zhou, Pin Zhou, Chuanliang Hu

**Affiliations:** ^1^Department of Spinal Surgery, Gaoyou Hospital Affiliated Soochow University, Gaoyou, Jiangsu 225600, China; ^2^Department of Orthopedics, Gaoyou People's Hospital, Gaoyou, Jiangsu 225600, China; ^3^Orthopedic Institute, Department of Orthopedics, The First Affiliated Hospital of Soochow University, Suzhou, Jiangsu 215007, China; ^4^Department of Orthopedics, Gaoyou Hospital of Integrated Traditional Chinese and Western Medicine, Gaoyou, Jiangsu 225600, China

## Abstract

**Objective:**

To investigate the effects of Danshen on the imaging and histological parameters, expression levels of ECM-associated proteins and inflammatory factors, and antioxidative activity in the degenerated intervertebral disc (IVD) of SD rats.

**Methods:**

Sixty male rats were randomly divided into three groups (control, IDD, and Danshen IDD). Percutaneous needle puncture in Co8-9 intervertebral disc was conducted in all rats of the IDD and Danshen IDD groups to induce intervertebral disc degeneration (IDD). After operation, animals of the Danshen IDD group were administrated with Danshen granules (3 g/kg body weight ) by gavage once a day. Four weeks later, the coccygeal vertebrae were harvested and used for imaging (disc height and MR signal), histological, immunohistochemical, and biochemical [water content, glycosaminoglycans (GAG), superoxide dismutase (SOD2), glutathione (GSH), and malondialdehyde (MDA)] analyses.

**Results:**

The puncture induced significant decreased IVD space and MR T2 signal at both 2 and 4 weeks, which were attenuated by Danshen treatment. The disc degeneration in the IDD group (HE and Safranin O-Fast Green histological staining was markedly more serious compared with that in the control group. Four weeks of Danshen treatment significantly alleviated this degeneration compared with the IDD group. Needle puncture resulted in the upregulation of IL-1*β* and TNF-*α*, MMP-3, and downregulation of COL2 and aggrecan in the IDD group. However, this change was significantly weakened by Danshen treatment. Significantly lower water and GAG content, as well as the SOD2 and GSH levels, in the IDD group were found compared with those in the control group. However, the above parameters of the Danshen IDD group were significantly higher than those of the IDD group. Danshen treatment significantly decreased the content of MDA which was increased by needle puncture in the IDD group.

**Conclusion:**

Danshen can attenuate intervertebral disc degeneration in SD rats by suppressing the oxidation reaction.

## 1. Introduction

With the aging of society, low back pain has become a common disease that plagues the physical and mental health of middle-aged and elderly people [1]. Intervertebral disc degeneration (IDD) is believed to be one of the main causes of low back pain and its etiology is complex and multifactorial, including aging, mechanical stress, smoking, infection, trauma, and genetics [2]. Intervertebral disc (IVD) primarily contains three parts: nucleus pulposus (NP), annulus fibrosus (AF), and cartilage endplate (EP). Structural or functional abnormalities of any component can finally result in IDD. The degenerated IVD shows a reduction in intervertebral height, the appearance of annulus fissures, the dysfunction of NPCs, the loss of ECM and water, and the calcification of the vertebral endplates, as well as increased production of inflammatory factors such as interleukin-1*β* (IL-1*β*) and tumor necrosis factor-*α* (TNF-*α*) [3]. At present, the specific mechanism of IDD is still not clear. However, increasing evidences demonstrate that oxidative stress might play a crucial role in the occurrence and development of IDD [4–6].

According to the free radical theory of aging, the decline of tissue and organ function is closely related to the oxidative stress caused by reactive oxide species (ROS) [7]. Excessive ROS can inhibit normal cellular activity by destroying cell lipid, protein, and DNA content [8]. It has been reported that malondialdehyde (MDA) and the peroxidation product of polyunsaturated fatty acid residues accumulate in degenerated IVDs of rats [9]. In short, in the process of IDD, systemic and local oxidative stress increases significantly, indicating that oxidative stress may play a crucial role in the pathological development of IDD. MDA, SOD2, and glutathione (GSH) are indicators commonly used to evaluate the oxidative stress of the body, and their levels can reflect the severity of the oxidative stress damage. As the final decomposition product of cell membrane lipid peroxidation, MDA can destroy the structure and function of cell membrane, finally inducing cell senescence or death [10, 11]. SOD2 is an important antioxidant enzyme, which is widely distributed in various organisms and has the function of resisting oxidative stress. GSH is an important antioxidant, which is essential in the regulation of protein disulfide bonds and the treatment of electrophiles and oxidants. The determination of the GSH concentration is indispensable in many studies on oxidative stress [12, 13].

The common treatments of IDD contain conservative treatment and surgical treatment. Bed rest, nonsteroidal anti-inflammatory drugs, analgesia, and physical therapy are common conservative treatments, which can relieve pain to a certain extent, but cannot prevent the pathological progression of IDD. When conservative treatment fails, surgical treatment becomes the first choice. However, surgical treatment is expensive and traumatic, and some patients still have poor recovery after surgery.

Danshen (Salvia miltiorrhiza), a traditional Chinese medicine, can promote blood circulation and remove blood stasis. Danshen has a wide range of pharmacological effects and is primarily used clinically for the treatment of irregular menstruation, palpitations, insomnia, and various cardiovascular diseases, especially angina pectoris and myocardial infarction [14]. In recent years, a number of researches have confirmed the effects of Danshen on improving microcirculation, anticoagulant, antithrombotic, antihypertensive, and antioxidant [15], which has broad application prospects. Although the specific antioxidant mechanism is unknown, Danshen has been proved to act as a ROS inhibitor [16, 17]. Zhang et al. [18] found that Danshen has a significant protective effect on H_2_O_2_-induced H9c2 cardiomyocyte apoptosis at low concentrations. Comparative studies with vitamin C show that Danshen can scavenge more than 90% of free radicals and can be used to prevent cell damage caused by free radicals and intracellular ROS [19]. To sum up, Danshen has been proved to be functional in different cell types via the antioxidation process as an adjuvant treatment and the use of Danshen to potentially alleviate disorders has been investigated.

However, whether Danshen can inhibit the development of IDD via antioxidation remains unknown. Therefore, we hypothesized that Danshen can alleviate intervertebral disc degeneration through its antioxidant effect. This study is aimed at exploring the effects of Danshen on the imaging and histological parameters, water and GAG content, expression levels of ECM-associated proteins, and inflammatory factors, as well as SOD2, GSH, and MDA level of degenerated IVDs in a percutaneous puncture SD model.

## 2. Methods and Materials

### 2.1. Animals

Sixty 12-week-old Sprague Dawley rats (30 males and 30 females) were used. All animal experiments were approved by the Animal Care and Experiment Committee of Soochow University. All rats were group housed under a 12 h light/dark cycle and had free access to a standard diet and sterile water.

### 2.2. IDD Model and Experimental Design

All 60 animals were randomly divided into three groups (control, IDD, and Danshen IDD group), with 20 animals in each group. After one week of acclimatization, all rats were anesthetized by inhalation of 2% fluothane in oxygen/nitrous oxide. The needle puncture model was performed in the Co8-9 IVD of all rats in IDD and Danshen IDD groups for inducing the IDD model as previously described [20, 21]. The Co8-9 tail IVD was punctured using a 20-gauge needle with full penetration. Penicillin was injected into the operational animals to prevent postoperative infection. All procedures were approved by our animal welfare committee. Danshen granules were purchased from Shanghai First Biochemical Pharmaceutical Co., Ltd. (Shanghai, China). After surgery, the rats in the Danshen IDD group were administrated with a certain amount of Danshen granules (3 g/kg body weight dissolved in distilled water) by gavage once a day over a 4-week period according to the dose used in previous reports [22]. The rats in the control and IDD groups were treated with only distilled water. All steps of this study complied with the Animal Research Reporting In Vivo Experiments (ARRIVE) Guidelines for reporting animal research.

### 2.3. Tissue Preparation

All animals in three groups were euthanized by an excess of isoflurane (RWD Life Science Co., Shenzhen, China) four weeks after the operation. The whole discs of the punctured segments (Co8-9) in all rats were surgically removed and dissected. Ten discs from each group were fixed for 48 h in 4% paraformaldehyde (Beyotime BioTechnology Co. Ltd., Shanghai, China) at 4°C then decalcified in 10% EDTA (Biosharp, Hefei, China) in 0.01 M PBS for 1 month at 4°C. The decalcified specimens were then embedded (Leica, Richmond, USA) in paraffin for further histological staining and immunohistochemistry. The remaining ten discs of each group were removed in liquid nitrogen after being harvested and stored at -80°C for western blot analysis and biochemical analysis. Histopathology, histological score, water and GAG content, expression levels of ECM-associated proteins and inflammatory factors, and GSH, MDA, and SOD2 concentration of the IVD were evaluated.

### 2.4. Radiographic Analysis and Magnetic Resonance Imaging (MRI) Examination

At two and four weeks after the needle puncture, all rats underwent X-ray radiography and MRI scans under isoflurane anesthesia. Radiographic images were taken with a digital X-ray machine (SHI- MADZU, Japan), and images were stored in a digital form. Based on the previously reported method [23], the disc height index (DHI) was calculated by averaging the measurements obtained from the anterior, middle, and posterior portions of the disc height and dividing them by the average height of the adjacent vertebral body using the image analysis program ImageJ. Changes in the DHI (representing the disc space) of IVD were presented as %DHI and normalized to DHI of preoperative IVD (%DHI = (DHI postlesion/DHI prelesion)∗100).

T2 mapping magnetic resonance imaging (MRI) sequence is usually used to reflect the water proton molecule movements in the extracellular matrix of collagen and proteoglycan [24]. At the termination of the study, the IVDs of all rat coccygeal vertebrae were scanned in a 1.5 T MRI scanner (GE, USA). The T2 signal intensities were calculated using image analysis program ImageJ to indirectly measure the extent of disc hydration. The mean T2 signal intensity in the control IVD was set as reference for that of the punctured disc in each rat. Therefore, the normalized disc intensity was presented from 0 to 1.

### 2.5. Histological Evaluation

For histological analysis, 5 *μ*m serial sections were prepared from the midsagittal region. The histological slices were stained with hematoxylin and eosin (H&E), Safranin O-Fast Green, and Alcian blue according to the standard procedures to reveal histological changes in the IVD tissues of different groups. Histological evaluation of IVD was performed based on a grading system reported by Han et al. [20]. The grading system was calculated based on the cellularity and morphology of the annulus fibrosus (AF), nucleus pulposus (NP), and the border between the AF and NP using a scale with five categories of IDD changes with scores ranging from 5 points (representing a normal disc) to 15 points (representing a severely degenerated disc) [20].

### 2.6. Immunohistochemical Analysis

Immunohistochemical analysis was conducted on decalcified sections of Co8-9 IVD under standard procedures [25]. ECM-associated proteins collagen II (COL2), aggrecan, and matrix metalloproteinase-3 (MMP-3) and inflammatory factors IL-1*β* and TNF-*α* in the IVDs were semiquantitatively analyzed. The sections were incubated with rabbit primary antibodies (COL2 1 : 100, aggrecan 1 : 100, MMP-3 1 : 100, IL-1*β* 1 : 200, and TNF-*α* 1 : 200; Abcam, England) or control rabbit IgG (1 : 100 in 5% BSA) overnight at 4°C and goat anti-rabbit secondary antibody (Cell Signaling Technology, USA, dilution 1 : 400). After being visualized by diaminobenzidine-based peroxidase (DAB) substrate, the images were acquired using an Olympus light microscope (Japan) at 40x magnification. Immunohistochemical staining results were further analyzed using a semiquantitative method, as previously described [25]. Each slice was observed in ten vision fields. The number of stained positive cells and their staining intensity were used for scoring, and the two scores (range: 0–12) were multiplied to obtain protein expression intensity. All sections were analyzed by two independent observers blinded to the experimental details. There was no significant difference within intraobserver and interobserver measurements.

### 2.7. Western Blot Analysis

The expression levels of COL2, aggrecan, and MMP-3 in each IVD were examined by a Western blot method. The concentration of isolated proteins was analyzed by a BCA Assay kit (Beyotime BioTechnology Co. Ltd., Shanghai, China). Equal quantities of protein from each sample were subsequently separated by gel electrophoresis and transferred onto polyvinylidene difluoride membranes. After blocking with 5% nonfat milk in Tris-buffered saline (TBS) containing 0.1% Tween-20 (TBST) for 1 h, the membrane was incubated with anti-rat primary antibodies in 5% nonfat milk diluted in TBST at 4°C overnight: COL2 (1 : 1000), aggrecan (1 : 1000), MMP-3 (1 : 1000), and GAPDH (1 : 2000). Then, the membranes were incubated with the HRP-conjugated secondary antibodies for 1 hour at 37°C. The membrane bands were detected by an enhanced chemiluminescent system, and the intensity of bands was analyzed by ImageJ software (NIH, USA). Finally, the relative optical densities of all bands were normalized to GAPDH.

### 2.8. Biochemical Analysis

After being removed from the liquid nitrogen, all tail discs were immediately dissected from their surrounding soft tissues carefully. Wet weight (WW) of the IVD tissue was immediately obtained after dissection. Then, the tissues were dehydrated at 60°C for one day and tissue dry weight (DW) was obtained, as described previously [20]. Thus, the water content of each disc was shown as follows: %H_2_O = 100∗(WW − DW)/WW. Next, the discs in each group were digested by papain (Beyotime BioTechnology Co. Ltd., Shanghai, China) in 1.5 mL of 20 mM sodium phosphate buffer at 65°C for 1 hour. The GAG concentration of IVD was measured based on the previously described method [26]. The DW protein content of each IVD was used to standardize the GAG content. The GSH content was measured using the Glutathione Assay Kit purchased from Beyotime BioTechnology Co. Ltd. according to the standard experimental procedure. The GSH concentration was assayed based on the standard curve of GSH. Lipid Peroxidation MDA Assay Kit (Beyotime BioTechnology Co. Ltd., Shanghai, China) was used to calculate the MDA content in IVDs of all animals. The sandwich ELISA method was employed to detect the SOD2 using Superoxide Dismutase Assay Kit (Beyotime BioTechnology Co. Ltd., Shanghai, China), and SOD2 levels were measured by comparing the standard curve in photometry according to the manufacturer's protocol.

### 2.9. Statistical Analyses

Experimental data were expressed as the means ± standard deviation (SD). All experiments were repeated at least three times. Differences among groups were assessed using one-way ANOVA after verification of normality. Post hoc comparisons between two groups were tested using a least square difference (LSD) method. Statistical analysis was performed using SPSS v17.0 software (SPSS Inc., Chicago, IL, USA). *P* values < 0.05 were considered statistically significant.

## 3. Results

### 3.1. Radiographic Assessment and MRI Examination

**Figure 1:**
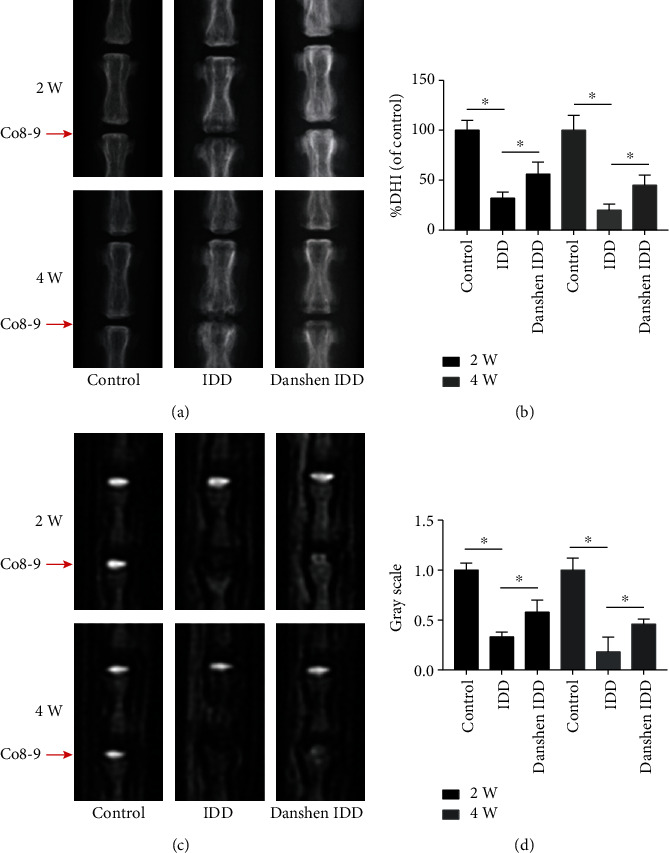
Imaging characteristics of IVDs in each group after 2 and 4 weeks. (a) Representative radiographs (X-ray) of the Co8-9 discs in the control group, IDD group, and Danshen IDD group. (b) Analysis of disc height index (DHI) based on X-ray film. A significant decrease of %DHI was observed in the IDD group. However, this reduction of disc space was significantly alleviated in the Danshen IDD group. (c) Representative images of T2-weighted MRI in Co8-9 discs. (d) Analysis of signal intensity based on MRI. Punctured discs in the IDD group showed a significant decrease of MRI signal intensity. However, the signal intensity in the Danshen IDD group was significantly higher compared to that in the IDD group. The values are expressed as the mean ± SD. ^∗^*P* < 0.05. IDD: intervertebral disc degeneration.

Radiographic assessment of the IVD height was performed at the 2- and 4-week time point using the calculated DHI by averaging measures before and after disc injury. Changes of IVD space at Co8-9 and the representative images of disc X-ray in the three groups are show in Figure 1(a). At 2 weeks after puncture, the IVDs in the IDD group indicated obvious signs of disc degeneration including significantly decreased IVD space and tissue swelling. In the Danshen IDD group, tissue swelling and height loss of the IVD were significantly alleviated compared to those in the IDD group. This finding indicated that Danshen treatment can protect the IVD from degeneration, which was further verified by the 4-week results and the subsequent MRI examination.

The T2-weighed MR signal intensity of IVDs can indicate the degeneration of the discs.  Figure 1(c) shows the representative images of tail disc in the three groups. Punctured Co8-9 IVDs in the IDD group showed a significant decrease of the T2-weighted signal in comparison with those in the control group at 2 weeks. The T2-weighed signal intensity in the Danshen IDD group was significantly higher than that of IDD group after 2-week Danshen treatment. Similar results were also found in the three groups at 4 weeks after puncture.

### 3.2. Histology

**Figure 2:**
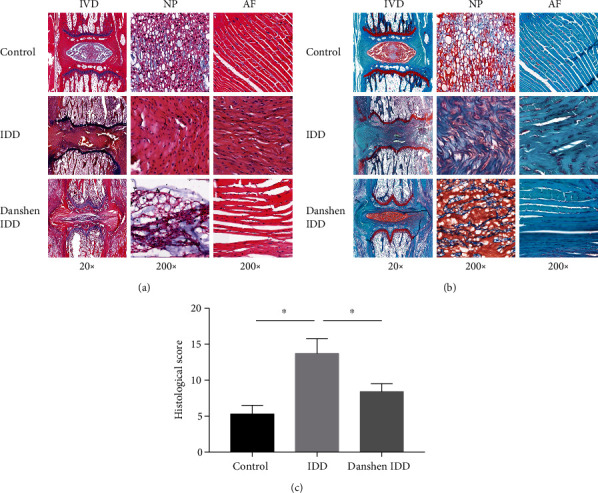
Histological analysis of IDD. (a) Representative histological appearance of IVDs stained with H&E. The control IVD presents a rounded nucleus pulposus, well-organized collagen lamellas, and a well-defined border between the annulus fibrosus (AF) and nucleus pulposus (NP). NP cells were stellar shaped and evenly distributed in the NP. In the IDD group, the size of the NP decreased and the border between the AF and NP became interrupted. The NP cells became large, rounded, clustered, and separated by dense areas of proteoglycan matrix. The annular collagen layers became disorganized with inward bulging of the inner annulus. The degenerated characteristics of rat IVD was alleviated by four weeks of Danshen treatment. (b) Representative histological appearance of IVDs stained with Safranin O-Green. (c) Analysis of the extent of IDD based on histological staining. Significantly higher histological scores were found four weeks after IDD induction. The histological score of the Danshen IDD group was significantly decreased than that of the IDD group. The values are expressed as the mean ± SD. ^∗^*P* < 0.05. IDD: intervertebral disc degeneration; IVD: intervertebral disc; NP: nucleus pulposus; AF: annulus fibrosus.

The HE and Safranin O-Fast Green staining results of rat tail IVD and representative histological appearance of three groups are shown in Figure 2. In the control group, tail discs contained a rounded NP, well-organized collagen lamellas, and a clear boundary between the AF and NP. Nuclear cells were stellar shaped and evenly distributed in the NP. Annular cells were mostly fibroblastic and located between well-organized collagen layers. However, in the IDD group, the discs demonstrated a typical disc degeneration features including the decreased size of the NP and the interrupted border between the NP and AF. The nuclear cells turned large and round and gathered to form clusters, sometimes separated by proteoglycan matrix. Inward bulging of the inner annulus appeared in disorganized collagen layers of AF. In the Danshen IDD group, the degenerated characteristics of rat IVD were alleviated by four weeks of Danshen treatment, including the appearance of partly preserved NP and regular arrangement of AF. (Figures 2(a) and 2(b)).

The histological evaluation of IVD is shown in Figure 2(c). Histology was shown significantly different in three groups (*P* < 0.001). The scores of IVD histological staining of both the IDD and the Danshen IDD groups were significantly higher than that of the control group (*P* < 0.001). However, the histological score of the Danshen IDD group was significantly lower in comparison with that of the IDD group (*P* < 0.001). Therefore, Danshen could significantly protect the structural integrity of rat IVD and delay IDD.

### 3.3. Water and GAG Content of IVD

**Figure 3:**
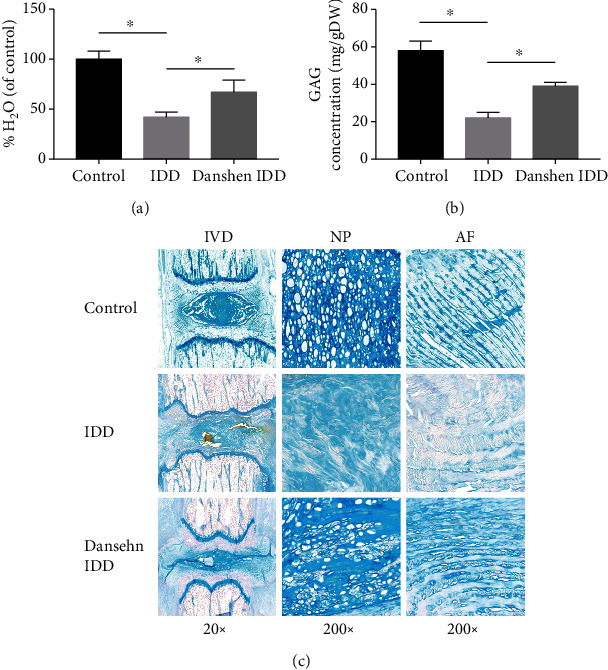
Analysis of water and GAG content of each group. (a) Water content of each group. (b) GAG content of each group. Significant decrease in water (a) and GAG (b) content in the IDD group was observed. However, this decrease was significantly alleviated by Danshen treatment in the Danshen IDD group. (c) Representative staining of Alcian blue staining of each group. The blue dye is proportional to the GAG content. The values are expressed as the mean ± SD. ^∗^*P* < 0.05. IDD: intervertebral disc degeneration; IVD: intervertebral disc; NP: nucleus pulposus; AF: annulus fibrosus.

The reduction of water content in the IVD is one of the typical signs of IDD. Our data demonstrated that disc needle puncture induced a significant decrease of the water content in the IDD group (Figure 3(a)). However, the water content of IVD in the Danshen IDD group was significantly higher compared with that in the IDD group with four weeks of Danshen treatment.

The GAG content in IVD can reflect its proteoglycan content. Interestingly, a similar pattern as water content was found in the amount of GAG in all tail IVDs in the three groups (Figure 3(b)). The GAG content of IVD in the IDD group was significantly decreased after four weeks of needle puncture. The GAG concentration in the Danshen IDD group was significantly higher than that of the IDD group with four weeks of Danshen treatment. Alcian blue is a commonly used dye for presenting GAG in tissue sections. In order to visualize the different contents of proteoglycan components of disc ECM, Alcian blue staining was performed, and the representative images are shown in Figure 3(c). The darkest blue staining was found in the IVD in the control group. Staining of the IDD group was shown to be lighter than that of the Danshen IDD group. These findings were consistent with that in quantitative analysis.

### 3.4. Expression Levels of COL2, Aggrecan, and MMP-3

**Figure 4:**
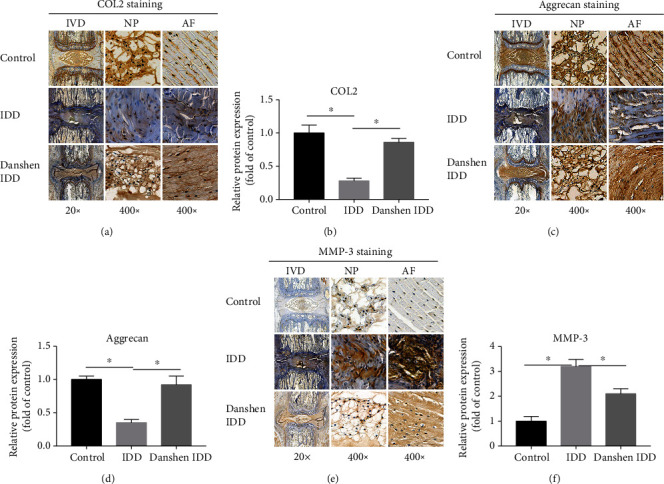
Immunohistochemical analysis of COL2, aggrecan, and MMP-3. (a) Representative images of COL2 staining. (b) Semiquantitative analysis of COL2. (c) Representative images of aggrecan staining. (d) Semiquantitative analysis of aggrecan. (e) Representative images of MMP-3 staining. (f) Semiquantitative analysis of MMP-3. Significant decrease in COL2 and aggrecan levels and increase in MMP-3 level in the IDD group was observed. However, this change was significantly alleviated by Danshen treatment. The values are expressed as the mean ± SD. ^∗^*P* < 0.05. IDD: intervertebral disc degeneration; IVD: intervertebral disc; NP: nucleus pulposus; AF: annulus fibrosus; COL2: collagen II; MMP-3: matrix metalloproteinase-3.

**Figure 5:**
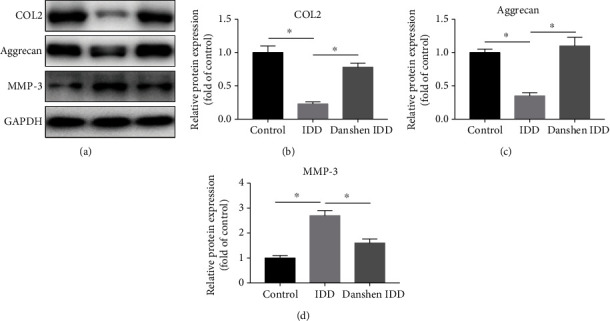
Western blot analysis of COL2, aggrecan, and MMP-3 in each group. (a) Protein expression levels of COL2, aggrecan, and MMP-3. (b) Semiquantitative analysis of COL2. (c) Semiquantitative analysis of aggrecan. (d) Semiquantitative analysis of MMP-3. The relative ratio of target proteins and GAPDH was calculated based on their gray values. Significant decrease in COL2 and aggrecan levels and increase in MMP-3 level in the IDD group were observed. However, this change was significantly alleviated by Danshen treatment. The values are expressed as the mean ± SD. ^∗^*P* < 0.05. IDD: intervertebral disc degeneration; COL2: collagen II; MMP-3: matrix metalloproteinase-3; GAPDH: glyceraldehyde-3-phosphate dehydrogenase.

In order to investigate the ECM remodeling of IVD, the immunohistochemical staining and western blot analysis of COL2, aggrecan, and MMP-3 were conducted. In the IDD group, the needle puncture resulted in the ECM remodeling by suppressing the production of COL2 and aggrecan and activating MMP-3, which were both shown in immunohistochemical analysis and western blot (Figures 4 and 5). After 4 weeks of Danshen treatment, the decreased expressions of COL2 and aggrecan were significantly restored and the increased MMP-3 level was concomitantly weakened in the Danshen IDD group. These results indicated that Danshen can protect the IVD from degeneration by regulating the components of NP ECM.

### 3.5. Immunohistochemical Staining of IL-1*β* and TNF-*α*

**Figure 6:**
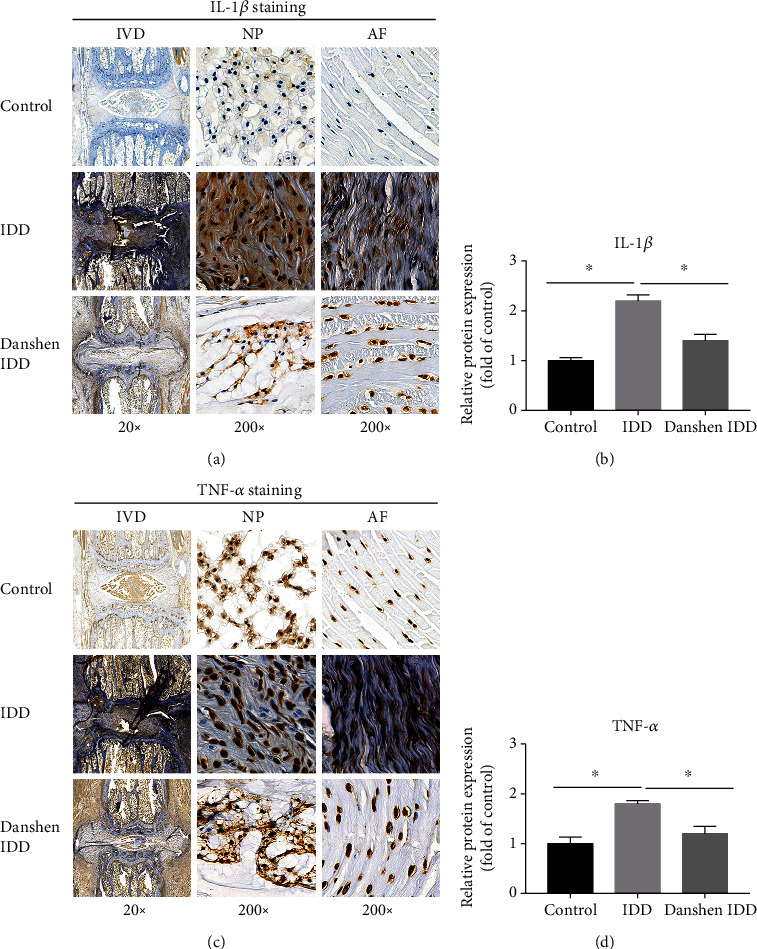
Immunohistochemical analysis of IL-1*β* and TNF-*α*. (a) Representative images of IL-1*β* staining. (b) Semiquantitative analysis of IL-1*β*. (c) Representative images of TNF-*α* staining. (d) Semiquantitative analysis of TNF-*α*. Significant increase in IL-1*β* and TNF-*α* level in the IDD group was observed. However, this increase was significantly downregulated by Danshen treatment. The values are expressed as the mean ± SD. ^∗^*P* < 0.05. IDD: intervertebral disc degeneration; IVD: intervertebral disc; NP: nucleus pulposus; AF: annulus fibrosus; COL2: collagen II; MMP-3: matrix metalloproteinase-3; IL-1*β*: interleukin-1*β*; TNF-*α*: tumor necrosis factor-*α*.

The expressions of inflammatory factors IL-1*β* and TNF-*α* in the IVDs were explored and are shown in Figure 6. The light yellow-stained IL-1*β* and TNF-*α* in the control group represented the low expression level of proteins, while brown-stained sections in the IDD group represented the high expressions levels. It appeared that these proteins were primarily distributed within the ECM of NP. Semiquantitative analysis showed that production of IL-1*β* and TNF-*α* in the IDD group was significantly promoted, which indicated that inflammatory response was activated during the IDD process. After Danshen treatment, the expression levels of IL-1*β* and TNF-*α* were significantly inhibited compared to the IDD group. This data indicated that Danshen can inhibit the inflammation during the IDD process.

### 3.6. GSH, SOD2, and MDA Concentrations of IVD

**Figure 7:**
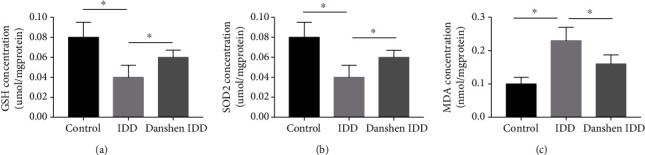
GSH, SOD2 and MDA content in tail IVDs of each group. Significant decrease in GSH (a) and SOD2 (b) content in IDD group was observed compared with the control group and this decrease was significantly alleviated by Danshen treatment. Significant increase in MDA (c) content in IDD group was observed compared with the control group. However, this increase was significantly alleviated by Danshen treatment in the Danshen IDD group. The values are expressed as mean ± SD. ^∗^*P* < 0.05. IDD: intervertebral disc degeneration; GSH: glutathione; SOD2: superoxide dismutase; MDA: malondialdehyde.

Figure 7(a) shows that the GSH contents of IVD in the three groups were significantly different. The GSH level in IVD in the IDD group (0.041 ± 0.013 *μ*mol/mg protein) was significantly lower compared to that in the control group (0.082 ± 0.024 *μ*mol/mg protein, *P* < 0.001). However, the GSH concentration of IVD in the IDD Danshen group (0.059 ± 0.015 *μ*mol/mg protein) was significantly higher than that of the IDD group after four weeks of Danshen medication (*P* < 0.001).

A similar pattern was also found in the SOD2 concentration of all tail IVDs in the three groups (Figure 7(b)). After four weeks of needle puncture, the SOD2 concentration of IVD in the IDD group was significantly decreased (*P* < 0.001). After four weeks of Danshen treatment, the SOD2 concentration in the Danshen IDD group (0.064 ± 0.011 *μ*mol/mg protein) was markedly higher compared to that in the IDD group (0.043 ± 0.014 *μ*mol/mg protein, *P* < 0.001).

MDA concentrations of tail IVD in the three groups were significantly different as shown in Figure 7(c). The MDA level in IVD in the IDD group (0.233 ± 0.042 nmol/mg protein) was significantly higher compared to that in the control group (0.109 ± 0.018 nmol/mg protein, *P* < 0.001). The MDA concentration of IVD in the IDD Danshen group (0.165 ± 0.035 nmol/mg protein) was markedly decreased compared to that in the IDD group (*P* < 0.001).

## 4. Discussion

At present, the most popular method is IVD injury in which the damage of AF, NP, or endplate through surgical approach leads to corresponding pathological changes and causes IDD finally [27]. Needle puncture is the most common way of damage models due to its reproducibility and the short period resulting in degenerative changes [28]. The rodents are proposed as an ideal model for IDD research because of easily degenerated tail discs of puncture [20]. In our research, the IDD model was induced by needle puncture with full penetration and the characteristic changes of IDD were shown, which demonstrated that the needle puncture model was quite effective.

In our in vivo research, a relative index %DHI (representing the IVD height) and MRI T2 signal strength were used to reflect the imaging alterations of IVDs. In the IDD group, significantly narrowed IVD space and tissue swelling was found at both 2 and 4 weeks, and the MR T2 signal of IVDs was obviously decreased and almost black. However, this IVD height and signal decrease were significantly attenuated by the Danshen treatment at both two time points. These findings indicated that Danshen could inhibit the change of disc height and MRI signal induced by dis injury, which was confirmed by the subsequent histological staining and protein expression analysis.

The HE and Safranin O-Fast Green histological staining and histological scores were used to assess the extent of tail disc degeneration in the three experimental groups. In the IDD group, needle puncture led to histological changes including the decreased NP size and the interrupted border between NP and AF, as well as nuclear cells gathering to form clusters and inward bulging of the inner annulus in disorganized collagen layers of AF. These altered morphological features were consistent with many of reports in rats [20, 29, 30] and human degenerative IVDs [23, 31]. Additionally, the histological score of the Danshen IDD group was lower than that of the IDD group, which demonstrates that Danshen treatment could prevent disc degeneration in rats.

Under physiological conditions, adequate GAG is crucial for the spine to withstand pressure and can help to expand discs and keep the separation of vertebrae [32]. The changes of water and GAG content in the IVDs may affect disc functions and be directly involved in IDD. In our research, the water and GAG content in the IDD disc was markedly lowered than that of the control IVD. Similar results were previously reported in which total GAG reduction of IVDs appeared at as early as the initial stage of IDD [33, 34]. After four weeks of Danshen treatment, the water and GAG content significantly increased, which was consistent with the recovery of MRI T2 signal in the Danshen IDD group. Our data suggested that Danshen might help to protect water and GAG from loss and to slow down the occurrence of IDD.

COL2 and aggrecan are two main components of the ECM and can keep the fluids within the IVDs and preserve the resilience and volume of NP. The MMP family, especially MMP-3, is the main catabolic enzyme that is responsible for the ECM degradation. The abnormal reduction of COL2 and aggrecan or increase of MMP-3 may result in the failure of the NP structure and ultimately the onset of IDD. In our study, needle puncture downregulated the expressions of COL2 and aggrecan and upregulated the MMP-3 level. This catabolic change was significantly inhibited by Danshen treatment. In addition, inflammation plays crucial roles in the initiation and progression of IDD. The inflammatory factors IL-1*β* and TNF-*α* were reported to be involved in many pathological processes, including oxidative stress, autophagy, ECM destruction, and cell apoptosis [35–37]. We found that IL-1*β* and TNF-*α* were both more expressed in IVD tissues of the punctured rats compared with the control rats. However, this increase of inflammatory factors was greatly suppressed by Danshen in contrast to the IDD group, indicating the potent anti-inflammatory effect of Danshen. Taken together, our study demonstrated that Danshen was effective in protecting the structure integrity of the NP and inhibiting inflammation, which in turn delayed the IDD process.

It has been reported that redox imbalance can lead to damage to cells and tissues via oxidative stress [38]. Previous researches demonstrated that oxidative stress was closely related to histological changes of IVD and development of IDD [5, 39] and treatments that target oxidative stress may alleviate the progression of IDD [4]. Recent studies have found that Danshen could prevent the onset of oxidative stress in the aorta and eyes of diabetic rats [22], suggesting that Danshen could have a negative effect on oxidative stress. For further investigating the specific mechanism of Danshen attenuating IDD, the GSH, SOD2, and MDA (primary biomarkers of oxidative stress) contents of IVD tissues were measured. Our study showed that the GSH and SOD2 concentrations of rat IVDs in the IDD group were significantly lower compared with those in the control group. Our finding was similar to other research in which the activity of antioxidative materials decreased in age-related degenerated IVDs in Wistar rats [39]. The reduction in GSH and SOD2 content of degenerated IDD means an increase in consumption or decrease in production of the antioxidant substances. Additionally, increased intracellular ROS levels and oxidative stress under IVD injury by needle puncture have been previously reported [4]. Combining previous results and our findings, we believe that the decreased concentrations of GSH and SOD2 in the degenerated IVDs of rats are probably due to increased consumption of GSH and SOD2 in order to fight with the excessive ROS from IVD injury. However, Danshen treatment significantly increased the GSH and SOD2 content of IVDs in the Danshen IDD group. Similar findings have been reported that 4-week Danshen treatment significantly raised GSH content in articular cartilage and synovium tissues of osteoarthritis rabbits [40]. These results demonstrated that Danshen could either induce GSH and SOD2 synthesis or directly scavenge excessive ROS generated during disc injury. MDA, a degradation product of peroxidized polyunsaturated fatty acids, is a common marker of oxidative stress [41, 42]. Previous studies showed that the MDA level in patients with lumbar IDD was markedly increased compared to that in the control group [9] and that serum and intervertebral MDA levels gradually increased with age in rats [39]. Similar results were also found in our research that MDA content in IVDs of the IDD group was statistically higher than that of the control group. However, Danshen treatment markedly downregulate the level of MDA in IVDs of the Danshen group. Taken together, our data indicated that lipid peroxidation occurred during IDD and Danshen can inhibit lipid peroxidation in the discs of rat tails.

There are some limitations that should be considered in our research. First, the pathogenesis environment of rat IVDs is not the same as human's and it is better to include human IVDs in the future. Second, only one time point (four weeks) was used to explore the effect of Danshen on IVDs and dynamic observation of this effect may be more convincing. Third, the precise mechanism of Danshen inhibiting oxidative stress is still unclear and needs further studies.

In conclusion, the present study indicated that Danshen could attenuate intervertebral disc degeneration and inhibit inflammation via antioxidation in puncture-induced SD rats. It has been suggested that the use of Danshen may have broad prospects in the medical treatment of intervertebral disc degeneration.

## Data Availability

The data used to support the findings of this study are available from the corresponding author upon request.
